# Novel Marine Phenazines as Potential Cancer Chemopreventive and Anti-Inflammatory Agents

**DOI:** 10.3390/md10020451

**Published:** 2012-02-16

**Authors:** Tamara P. Kondratyuk, Eun-Jung Park, Rui Yu, Richard B. van Breemen, Ratnakar N. Asolkar, Brian T. Murphy, William Fenical, John M. Pezzuto

**Affiliations:** 1 College of Pharmacy, University of Hawaii at Hilo, 34 Rainbow Drive, Hilo, HI 96720, USA; Email: kondraty@hawaii.edu (T.P.K.); eunjungp@hawaii.edu (E.-J.P.); 2 Department of Medicinal Chemistry and Pharmacognosy, College of Pharmacy, University of Illinois at Chicago, Chicago, IL 60612, USA; Email: ryu9@uic.edu (R.Y.); breemen@uic.edu (R.B.B.); btmurphy@uic.edu (B.T.M.); 3 Center for Marine Biotechnology and Biomedicine, Scripps Institution of Oceanography, University of California-San Diego, La Jolla, CA 92093, USA; Email: rasolkar@marronebio.com (R.N.A.); wfenical@ucsd.edu (W.F.)

**Keywords:** apoptosis, chemoprevention, inflammation, NFκB, phenazines, lavanducyanin

## Abstract

Two new (**1** and **2**) and one known phenazine derivative (lavanducyanin, **3**) were isolated and identified from the fermentation broth of a marine-derived *Streptomyces* sp. (strain CNS284). In mammalian cell culture studies, compounds **1**, **2** and **3** inhibited TNF-α-induced NFκB activity (IC_50_ values of 4.1, 24.2, and 16.3 μM, respectively) and LPS-induced nitric oxide production (IC_50_ values of >48.6, 15.1, and 8.0 μM, respectively). PGE_2_ production was blocked with greater efficacy (IC_50_ values of 7.5, 0.89, and 0.63 μM, respectively), possibly due to inhibition of cyclooxygenases in addition to the expression of COX-2. Treatment of cultured HL-60 cells led to dose-dependent accumulation in the subG1 compartment of the cell cycle, as a result of apoptosis. These data provide greater insight on the biological potential of phenazine derivatives, and some guidance on how various substituents may alter potential anti-inflammatory and anti-cancer effects.

## 1. Introduction

Modulation of molecular targets that control cancer development and progression is one approach for chemoprevention. For example, transcriptional regulation of NFκB has been intensely studied. Major cellular targets for NFκB are chemokines, immune receptors, adhesion molecules, stress response genes, regulators of apoptosis, transcription factors, growth factors, enzymes, and cell cycle regulators [1,2,3]. These include the anti-apoptosis genes *bcl-2* and *bcl-xl*, *cylcooxygenase (COX)-2*, *matrix metalloproteinase-9 (MMP-9)*, genes encoding adhesion molecules, chemokines, inflammatory cytokines, and cell cycle-regulatory genes [4,5,6]. In addition, the NFκB pathway is central in inducible nitric oxide synthase (iNOS) induction [7]. Activity of iNOS is tightly regulated and includes activation of IκB and mitogen-activated protein kinases (MAPKs) kinases [8,9,10]. In principle, agents that can suppress NFκB activation and iNOS have the potential of suppressing carcinogenesis [11].

In response to various external stimuli, such as proinflammatory cytokines, bacterial lipopolysaccharide (LPS), UV, ROS and phorbol ester, COX-2 is transiently elevated in certain tissues, and serves as an interface between inflammation and cancer [12,13]. Abnormally elevated COX-2 causes promotion of cellular proliferation, suppression of apoptosis, and enhancement of angiogenesis and invasiveness, which accounts for its oncogenic function [14]. Elevated levels of PGE_2_, generated by COX-2, mediate inflammatory responses [15]. In various types of human cancers, elevated PGE_2_ levels promote cell proliferation and tumor-associated neovascularization, and inhibit cell death, thereby favoring tumor growth [16]. 

An additional consideration is cellular homeostasis. Cancer may be viewed as an example wherein the normal mechanisms of cell cycle regulation are dysfunctional, with either an over proliferation of cells and/or decreased removal of cells [17]. In fact, suppression of apoptosis during carcinogenesis is thought to play a central role in the development and progression of some cancers [18,19]. 

We report here the isolation and biological evaluation of two new phenazine derivatives (**1** and **2**) as well as lavanducyanin (**3**) from a marine derived *Streptomyces*. Metabolites of this type have been isolated from terrestrial *Streptomyces*, *Pseudomonas*, and a variety of marine microorganisms [20,21,22,23,24]. These compounds are generally formed by microorganisms when they have stopped dividing. Metabolism is slow and it has been noted that phenazine-producing organisms survive longer in their natural environment compared to non-producing species [24]. It is likely that phenazine production serves to protect against other microorganisms and microbial competitors [25]. In addition to normal physiological function, various biological activities have been explored that might be of value for the treatment of human ailments [25,26,27]. For example, it has been shown that lavanducyanin strongly inhibits the growth of P388 and L1210 cells in culture [28]. Conversely, the potential of phenazines and lavanducyanin to promote cellular growth has been reported [29]. Growth promoting action was confirmed by the MTT assay, as well as an increase in cell number and DNA synthesis [30]. Lavanducyanin competitively inhibits 5-α-reductase derived from rat, dog and human prostate [31].

In earlier investigations [23,27], based on inhibition of NFκB activity and cellular proliferation, *Streptomyces* sp. strain CNS284 was selected for activity-guided fractionation. This resulted in the isolation of a diversity of phenazine derivatives, including some with modest activity against NFκB. Phenazines with bromine substituents were discovered to increased inhibitory activity; molecules with two bromine atoms were even more active, and bromine-substituted hydroxyphenazines demonstrated higher activity than their methylated analogues [23]. Simultaneously, these compounds induced quinone reductase 1 (NQ01, QR1) and inhibited quinone reductase 2 (NQ02, QR2) as well as iNOS. With QR1 and QR2, several of the phenazine derivatives displayed IC_50_ in the nanomolar range. In particular, 2,4-dibromo-1-hydroxyphenazine was found to activate QR1 and glutathione *S*-transferase (GST) in cell culture, but *in vivo* activity of the compound was low as a result of poor bioavailability [27]. We now report related pharmacological studies conducted with two new bromine containing terpenoid phenazines (**1** and **2**) and lavanducyanin (**3**) produced by the marine bacterium *Streptomyces* sp. (strain CNS284).

## 2. Results and Discussion

### 2.1. Phenazines Isolation

**Figure 1:**
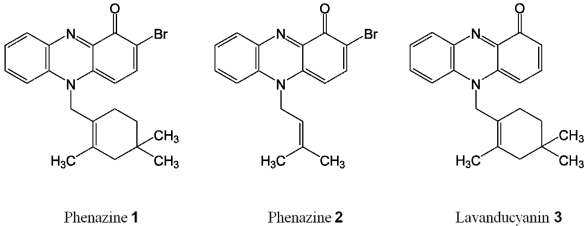
Chemical structures of phenazines **1**–**3**.

Fractionation of the fermentation broth of the marine-derived *Streptomyces* sp. (strain CNS284) resulted in the isolation of a complex mixture of brominated, terpenoid phenazines [32]. Among these were brominated terpenpoid phenazines **1** and **2** and also the known phenazine lavanducyanin (**3**) (Figure 1). All compounds belong to the terpenoid-substituted phenazines: compound **1**: N-substituted brominated monoterpene phenazine; compound **2**: N-substituted isoprenylated phenazine; compound **3**: lavanducyanin, an *N*-monoterpenoid first discovered and isolated from *Streptomyces* *aeriouvifer* [28,29].

### 2.2. Phenazines Inhibit TNF-α-Induced NFκB Activity

Activation of NFκB may promote cell proliferation or prevent cell death through apoptosis. Inhibition of NFκB signaling has potential application for the treatment or prevention of cancer; some inhibitors are known [33,34,35,36,37,38,39,40]. Using stably-transfected human embryonic kidney cells 293 (Panomics, Fremont, CA) that express a NFκB reporter when treated with tumor necrosis factor-α (TNF-α), treatment with phenazines **1**, **2** and **3** led to dose-dependent inhibition with IC_50_ values of 4.1 ± 0.3, 24.2 ± 2.6, and 16.3 ± 0.9 μM, respectively. Earlier we demonstrated that 2-bromo-1-hydroxyphenazine isolated from *Streptomyces* strain CNS284 and synthesized by a short and flexible route showed moderate inhibition of NFκB with an IC_50_ of 73 μM [23]. These results suggest inhibitory activity is influenced by the N-substituent pattern as well as bromination at C-2. With the same treatment protocol, positive controls *N*-tosyl-L-phenylalanyl chloromethyl ketone (TPCK) [39] BAY-11-7082 [40], and resveratrol yielded IC_50_ values of 30 ± 4.2, 2 ± 0.28 and 2.5 ± 0.3 µM, respectively. 

### 2.3. Transcriptional Regulation of iNOS and COX-2 by Phenazines

One of the key down-stream targets of NFκB is NOS, which catalyzes the oxidative deamination of L-arginine, producing NO, an important pro-inflammatory mediator [41]. Sustained high concentrations of NO contribute to carcinogenesis [42]. The bactericidal capacity of macrophages is in part mediated by the production of ROS and NO. In response to LPS and/or interferon-γ (IFN-γ), macrophages up-regulate iNOS, which catalyzes the oxidation of L-arginine to L-citrulline and NO. A second important down-stream target of NFκB is COX-2. This inducible form of cyclooxigenase generates prostaglandins, which are key players in the inflammatory response [15,43].

**Figure 2:**
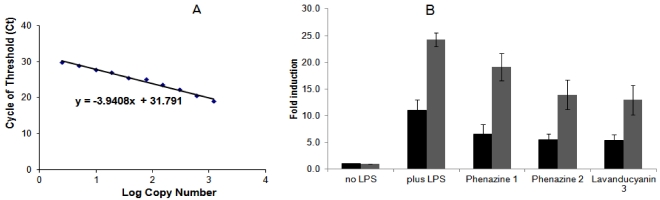
Effect of phenazines on *COX-2* (black) and *iNOS* (gray) mRNA expression in RAW 264.7 cells. Total RNA was isolated using the TRIZOL^®^ Reagent method (Invitrogen) from RAW 264.7 cells (2 × 10^5^ cells/well) after treatment with samples. cDNA was synthesized using the RT^2^ First Strand Kit (C-03, SA Biosciences) protocol. cDNA was used for quantitative real time PCRs with fluorescent Power SyBR^®^ Green PCR master mix (Applied Biosystems), employing *GAPDH*, *iNOS*, *COX-2* specific primers, and a 7300 Real Time PCR System (Applied Biosystems). The results were derived from two independent RNA preparations employing identical triplicates in each analysis and quantitated using *GAPDH* as the internal control, following the manufacturer’s instructions. (**A**) *GAPDH* standard curve for quantitation of *iNOS* and *COX-2* expression; (**B**) Levels of *COX-2* (black) and *iNOS* (gray) mRNA expression. The concentration and duration of treatment had no significant effect on the viability of Raw 264.7 cells.

As shown in Figure 2, LPS-treated RAW 264.7 cells were used as a model to assess the potential of phenazines **1**, **2** and **3** to inhibit the expression of *COX-2* and *iNOS*. Total mRNA samples were isolated, and cDNA was amplified with specific primers, using *GAPDH* as housekeeping gene. LPS induced the expression of *COX-2* and *iNOS* mRNA by approximately 11- and 24-fold, respectively. Co-incubation with phenazines **1**, **2** and **3** (50 µM) suppressed expression of *iNOS* mRNA by 21, 43, and 46% respectively, and *COX-2* expression by 40, 50 and 51%, respectively (Figure 2). These inhibitory results are not very discriminating, but do indicate transcriptional regulation, perhaps working in concern with NFκB.

### 2.4. Inhibition of NO and PGE_2_ Production in LPS-Induced RAW 264.7 Cells

Since the test phenazines (**1**–**3**) inhibited the NFκB signaling pathway, the effects on NO and PGE_2_ levels were determined with LPS-treated RAW 264.7 cells. As shown in Table 1, when treated with a fixed concentration of 50 µM, phenazines **2** and **3** inhibited NO production by over 90%. Dose-response studies yielded corresponding IC_50_ values of 15.1 and 8.0 µM. Phenazine **1** was less active with 40% inhibition of NO production at 50 µM and a high IC_50_ value. The highest concentration of phenazines (50 µM) was somewhat cytotoxic with RAW 264.7 cells, but this effect was eliminated at concentrations in a range close to the IC_50_ values. All three phenazines reduced LPS-induced PGE_2_ production in a dose-dependent manner, with IC_50_ values 7.15, 0.89, 0.63 µM, respectively. Resveratrol, used as a standard drug, inhibited the production of both NO and PGE_2_ with IC_50_ values of 31.9 and 2.5 µM, respectively. Previously it was shown that a phenazine compound lacking N-substitution (2-bromo-1-hydroxyphenazine) did not inhibit iNOS activity [23], so N-substitution plays an important role in the inhibition of NO production.

**Table 1:** The effect of phenazines **1**–**3** on LPS-induced NO and PGE_2_ production in RAW 264.7 cells.

Compound	NO	NO	NO	PGE_2_	PGE_2_
	% inhibition (50 µM)	% cell survival	IC_50_, µM	% inhibition (50 µM)	IC_50_, µM
Phenazine **1**	40.1 ± 5	68.9 ± 3.8	>48.6	72.5 ± 16.3	7.15 ± 2.03
Phenazine **2**	98.1 ± 0.5	56.0 ± 5.1	15.1 ± 2.7	86.9 ± 12.9	0.89 ± 0.22
Lavanducyanin **3**	97 ± 0.7	58.3 ± 4.6	8.0 ± 0.39	80.6 ± 18.4	0.63 ± 0.16
Resveratrol	93.0 ± 0.8	86.9 ± 3.9	31.9 ± 1.8	70.2 ± 13.9	2.5 ± 0.43

### 2.5. Direct Inhibition of COX-1 and COX-2 Activity

As described above, phenazines **2** and **3** strongly reduce the production of PGE_2_ with LPS-treated RAW 264.7 cells, but this does not directly correlate with the potency of inhibiting NFκB activity or COX-2 expression. Radical scavenging is one feature of phenazines [24]. However, based on the 2,2-diphenyl-1-picrylhydrazyl (DPPH) radical assay, none of the three phenazines demonstrate inhibition of greater than 15% when tested at a concentration of 200 μg/mL (data not shown). Another possible explanation could be the direct inhibition of cyclooxygenase. As shown in Table 2, all three phenazines inhibited both enzymes with IC_50_ values in µM range. The IC_50_ of phenazine **1** was approximately 3-fold lower with COX-2 than COX-1, but the IC_50_ values were not sufficient to explain the dramatic reduction of PGE_2_. Indomethacin was used as the positive control for ovine COX-1 (IC_50_ 0.42 ± 0.21 µM) and Celebrex^®^ (celecoxib) was used as the positive for human COX-2 (IC_50_ 0.05 ± 0.03 µM).

**Table 2:** Phenazines inhibit ovine COX-1 and human COX-2 activities.

	COX-1, IC_50_, µM	COX-2, IC_50_, µM	Selectivity COX-1/COX-2
Phenazine **1**	11.0 ± 0.53	4.0 ± 0.41	2.75
Phenazine **2**	5.6 ± 0.61	7.2 ± 0.13	0.78
Lavanducyanin **3**	30.0 ± 1.08	34.0 ± 1.1	0.88
Indomethacin	0.42 ± 0.21	ND	
Celecoxib	ND	0.05 ± 0.03	

ND: not determined.

### 2.6. Phenazines Induce Apoptosis

**Figure 3:**
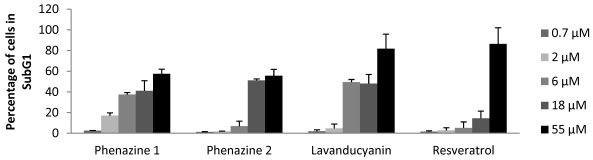
NIM-DAPI cell cycle analysis of HL-60 cells. HL-60 cells (2 × 10^5^ cells/well) were treated with different concentrations of the indicated compounds for 24 h. The media were discarded, and 4',6-diamidino-2-phenylindole solution (NIM-DAPI; Beckman Coulter) was added just before the measurement using a Cell Lab Quanta™ SC (Beckman Coulter) flow cytometer. NIM-DAPI-stained cells were analyzed after excitation. The distribution of cells in each phase of cell cycle was exhibited in a DNA histogram and percentage in subG_1_ was analyzed. Results are representative of two experiments in triplicate.

Apoptosis is a protective mechanism against the development of tumors. It eliminates damaged cells that may be wrongly induced to proliferate by different stimuli. When a cell reaches the G1 check-point, it can enter S phase or, if there is evidence of damage, it can initiate the apoptotic process [44]. Apoptosis can be induced by physiological activators, oncogenes, chemotherapeutic drugs, and ultraviolet or gamma radiation [45]. As shown in Figure 3, after a 24 h incubation period with various concentrations of phenazines **1**, **2** and **3**, HL-60 cells accumulated in the subG1 phase of the cell cycle, indicative of apoptosis. This was confirmed by using a Phoenix Flow Systems, Inc. APO-BRDU™ kit, a two color TUNEL (Terminal deoxynucleotide transferase dUTP Nick End Labeling) assay for labeling DNA breaks and total cellular DNA (data not shown). The results suggest all three phenazines are active in this process, with phenazine **3** demonstrating the greatest potency, followed by **1** and then **2**. Resveratrol was applied as a positive control.

## 3. Experimental Section

### 3.1. Cell Lines and Reagents

Methanol (HPLC grade), ethyl acetate (HPLC grade), marine broth, actinomycetes agar, cyclohexamide, agar, dimethyl sulfoxide (DMSO), lipopolysaccharide (LPS), sulfanilamide, 0.1% *N*-(1-naphthyl)ethylenediamine, sulforhodamine B (SRB), tetradecanoylphorbol 13-acetate (TPA), 2,2-diphenyl-1-picrylhydrazyl (DPPH), non-essential amino acids, and thiazolyl blue tetrazolium bromide (MTT) were purchased from Sigma-Aldrich, Inc. (St. Louis, MO, USA). Dulbecco’s Modified Eagle’s Medium (DMEM), antibiotic-antimycotic, Hygromycin B, MEM, sodium pyruvate, and Roswell Park Memorial Institute (RPMI) 1640 medium, were purchased from Invitrogen (Carlsbad, CA, USA). Reporter Lysis Buffer Luciferase Assay System was purchased from Promega (Madison, WI, USA). Tumor necrosis factor-α (TNF-α) was purchased from Calbiochem (Gibbstown, NJ, USA). All other chemicals were purchased from commercial sources and were of the highest grade and purity.

### 3.2. NFκB Assay

We employed human embryonic kidney cells 293 Panomic (Fremont, CA, USA) for monitoring changes occurring along the NFκB pathway. Stable constructed cells were seeded into sterile 96-well plate at 20 × 10^3^ cells per well. Cells maintained in Dulbecco’s modified Eagle’s medium (DMEM) Invitrogen Co. (Carlsbad, CA, USA), supplemented with 10% FBS, 100 units/mL penicillin, 100 µg/mL streptomycin, 2 mM L-glutamine. After 48 h incubation, the medium was replaced and cells were treated with various concentration of test substances dissolved in PBS. TNF-α (Human, Recombinant, *E. coli*, Calbiochem, Gibbstown, NJ, USA) was used as an activator at a concentration of 2 ng/mL (0.14 nM). The plate was incubated for 6 h. Spent medium was discarded and the cells were washed once with PBS. Cells were lysed using 50 µL (for 96-well plate) Reporter Lysis Buffer from Promega, by incubating for 5 min on a shaker, and stored at −80 °C. The luciferase assay was performed using the Luc assay system from Promega (Madison, WI, USA) [46]. The gene product, luciferase enzyme, reacts with luciferase substrate, emitting light which was detected using a luminometer (LUMIstar Galaxy BMG). Data for NFκB constructs are expressed as IC_50_ values (*i.e.*, concentration required to inhibit TNF-activated NFκB activity by 50%). As a positive control, two NFκB inhibitors were used: TPCK, IC_50_ = 3.8 µM and BAY-11, IC_50_ = 2.0 µM.

### 3.3. Nitric Oxide (NO) Synthase Assay

NO has a short half-life and is subsequently oxidized to the stable end product nitrite. The amount of nitrite was measured using the Griess reagent to assess the NO production [47,48]. RAW 264.7 cells (1 × 10^5^ cells/well) were incubated in a 96-well culture plate for 24 h. The cells were treated with various concentrations of compounds dissolved in phenol-red free DMEM for 30 min followed by 1 μg/mL of LPS treatment for 24 h. Nitrite in the media from cultured macrophages in each well was reacted with the Griess reagent [1:1 mixture (v/v) of 1% sulfanilamide in 2.5% H_3_PO_4_ and 0.1% *N*-(1-naphthyl)ethylenediamine in 2.5% H_3_PO_4_] and the absorbance was measured at 540 nm. The standard curve was created by using known concentrations of sodium nitrite. 

### 3.4. Cell Viability Assay

The cytotoxicity of the test substances (**1**–**3**) toward different cancer cells was determined as described previously [49,50,51]. Briefly, various concentrations of test compounds in DMSO were transferred to 96-well plates and incubated for 72 h at 37 °C in a CO_2_ incubator. The incubation was ended by the addition of trichloroacetic acid. The cells were then washed, air-dried, stained with SRB solution, and optical densities were determined at 515 nm using a ELx800NB Universal Microplate Reader, Bio-Tek Instruments. In each case, a zero-day control was performed by adding an equivalent number of cells to several wells, incubating at 37 °C for 30 min, and processing as described above. Percent cell survival was calculated using the formula: 

Percent cell survival = (OD_cells+tested compound_ − OD_day 0_)/OD_cells+10% DMSO_ − OD_day 0_) × 100

### 3.5. Determination of PGE_2_

RAW 264.7 cells (1 × 10^5^ cells/well, 200 µL) were incubated in a 96-well culture plate in the presence of 5% CO_2_ at 37 °C for 24 h. The medium was changed to non-colored DMEM containing 5% FBS, and the cells were treated with various concentrations of compounds for 30 min followed by 1 μg/mL of LPS treatment for 24 h. Cell media were collected, diluted 18-times and used for measuring PGE_2_. Nunc-Immuno 96-well plates were coated with secondary antibody (goat anti-mouse IgG) affinity purified (Jackson Immuno Research Laboratories). Aliquots of cell media were added to the immune plate with primary PGE_2_ monoclonal antibody (Cayman Chemical) and a tracer PGE_2_-acetylcholinesterase (Cayman Chemical) and incubated overnight at room temperature in the dark. The next day, the wells were aspirated, washed, 200 µL of Elman’s reagent was added in each well, and incubated 2–5 h at 37 °C out of direct light until control wells reach an optical density 0.4–0.5. Gentle rotating/shaking was used to decrease the time required for color development. Absorbance was read at 412 nm [52]. The PGE_2_ concentration of each sample was calculated from a PGE_2_ standard curve (PGE_2_ from Cayman Chemical). The percent inhibition as a function of the inhibitor concentration was graphed to determine the IC_50_ values (concentration at which there was 50% inhibition). 

### 3.6. COX-1 and COX-2 Inhibition Assays

Each COX reaction was initiated by adding 20 µL of arachidonic acid in Tris-HCl (pH 8.0) buffer to give a final concentration of 5 µM. The reaction was terminated after 2 min by adding 20 µL of 2.0 M HCl. The surrogate standard PGE_2_-d4 was added to correct for errors or degradation during sample handling and for variation in injection volume or instrument response during LC-MS/MS. After 30 min, PGE_2_ and its surrogate standard were extracted from each incubation mixture using 800 µL of hexane/ethyl acetate (50:50, v/v). The organic phase was removed, evaporated to dryness, and reconstituted in 100 µL methanol/water (50:50, v/v) for analysis using LC-MS/MS with an Applied Biosystems AP4000 triple quadrupole mass spectrometer as described previously [53,54]. The concentration of PGE_2_ in each sample was measured using LC-MS/MS, and the percent of COX inhibition by each test solution was determined by comparing the amount of PGE_2_ produced in the experiment with that produced in the negative control incubation. For IC_50_ value determination, 12 different concentrations of each inhibitor were assayed three times. The IC_50_ value of each inhibitor toward COX-1 or COX-2 was determined by plotting and analyzing the inhibition curve data using Graph Pad Prism 5 software [55]. The selectivity of each inhibitor was calculated as the ratio of the IC_50_ values (COX-1/COX-2) [53]. 

### 3.7. Cell Cycle Analysis

HL-60 cells (2 × 10^5^ cells/well) were treated with samples for 24 h. The media were discarded and nuclear isolation medium (4',6-diamidino-2-phenylindole, NIM-DAPI; Beckman Coulter) was added just before the measurement using a Cell Lab Quanta™ SC (Beckman Coulter) flow cytometer. NIM-DAPI-stained cells were analyzed after excitation. The distribution of cells in each phase of cell cycle was exhibited in a DNA histogram and percentage in subG_1_ was analyzed [56].

### 3.8. Detection of Apoptosis

HL-60 cells at a concentration of 2 × 10^6^ cells/mL were incubated with testing phenazines at different concentrations for 24 h. The cells were fixed with 1% paraformaldehyde in PBS and treated with ethanol to permiabilize the cells. Positive and negative control cells were provided already fixed. The DNA labeling reaction was carried out at 22–24 °C overnight. This assay was run on a Cell Lab Quanta™ SC, Beckman Coulter flow cytometer equipped with a 488 nm Argon laser as the light source. Propidium Iodide (total cellular DNA) and Fluorescein (apoptotic cells) were used. Propidium Iodide (PI) fluoresces at about 623 nm and Fluorescein at 520 nm when excited at 488 nm. Following the manufacturer’s instructions, an APO-BRDU™ Kit (Phoenix Flow Systems, Inc.) with a two color TUNEL (Terminal deoxynucleotide transferase dUTP Nick End Labeling) capability for labeling DNA breaks and total cellular DNA to detect apoptotic cells was used [57]. 

### 3.9. Real Time Quantitative PCR

RAW 264.7 cells (2 × 10^5^ cells/well) were treated with test samples and total RNA was isolated with TRIZOL^®^ Reagent (Invitrogen). RNA concentrations were measured using Nanodrop (ND 1000 V.3.1.0) and partially dissolved RNA samples had an A260/280 ratio <1.6. cDNA was synthesized using a RT^2^ First Strand Kit (C-03, SA Biosciences) protocol. cDNA was used in quantitative real time PCRs, using fluorescent Power SyBR^®^ Green PCR master mix (Applied Biosystems) and 7300 Real Time PCR System (Applied Biosystems). The results were derived from two independent RNA preparations employing identical triplicates in each analysis and quantified using *GAPDH* as the internal control, following the manufacturer’s instructions. The fluorescence intensity (*Rn*) corresponding to the cycle of threshold value (*Ct*) is used to quantitate a given mRNA, employing the *GAPDH* standard curve (Figure 3A). The equation Y = −3.9408 X + 31.791 was used to quantify the relative mRNA content, where Y represents the number of cycles corresponding to the *Ct* (*i.e.*, fluorescence intensity for a given mRNA species), and X represents the log copy number (*C0*) from which the relative content of mRNA was quantitated. PCR was performed on the cDNA using the following sense and antisense primers (Invitrogen) [58,59].

*GAPDH*

Forward: 5′-ACA GTC AGC CGC ATC TTC-3′,

Reverse: 5′-GTC CTT CCA CGA TAC CA-3′.

*β-Actin*

Forward: 5′-GCT ACA GCT TCA CCA CCA CAG-3′,

Reverse: 5′-GGT CTT TAC GGA TGT CAA CGT C-3′.

*COX-2*

Forward: 5-′GAA GTC TTT GGT CTG GTG CCT G-3′,

Reverse: 5′-GTC TGC TGG TTT GGA ATA GTT GC-3’.

*iNOS*

Forward: 5′-GGA GCG AGT TGT GGA TTG TC-3′,

Reverse: 5′-GTG AGG GCT TGG CTG AGT GAG-3′.

### 3.10. Evaluation of Antioxidant Capacity

To evaluate antioxidant capacity, 2,2-diphenyl-1-picrylhydrazyl (DPPH) free radical scavenging was performed according to the method of Lee *et al*. [60]. Briefly, 95 µL of DPPH radical solution (316 µM) was added in a 96-well plate containing 5 µL of each compound dissolved in 100% DMSO, and incubated for 30 min at 37 °C. The absorbance of each well was measured at 515 nm using a microplate reader. The DPPH radical scavenging activity of each sample was evaluated by calculating % of inhibition as follows: 

% inhibition = (1 − A_sample_/A_control_) × 100

### 3.11. Statistical Analysis

The results were expressed as the mean ± SD of triplicate experiments. Statistically significant values were compared using Student-Newman-Keuls and *p*-values less than 0.05 were considered statistically significant. 

## 4. Conclusions

Natural products have proven to be a useful source of cancer therapeutic and chemopreventive agents. Thus far, terrestrial plants have been most widely studied, with the marine environment remaining largely unexplored. In previous studies, the chemotherapeutic potential of lavanducyanin (**3**) was investigated [28,29,30,31]. We now report the ability of this substance to inhibit the activity of NFκB, COX-1 and COX-2, as well as the production of NO and PGE_2_. In addition, and perhaps as a consequence, cellular apoptosis is induced.

Beyond lavanducyanin, two new brominated terpenoid phenazines are reported along with their corresponding biological activity. Consistent with previous results [23], bromination at position-2 (phenazine 1) enhanced NFκB inhibitory activity by approximately 4-fold. This did not correlate with a greater reduction of NO or PGE_2_ production, but the compound did appear to induce apoptosis with greater efficacy, and inhibition of the catalytic activity of COX-2 was enhanced nearly 10-fold.

On the other hand, potent (sub-µM) inhibition of PGE_2_ production was observed with phenazine **2** and lavanducyanin (**3**). Additional definition of the mechanism of this response would be of interest since a strong correlation with the other activities monitored in this report was not apparent. This could be unique. In addition, the compounds are of interest due to the potential of modulating multiple targets. Although activity is generally not highly potent, this is not a prerequisite for effective chemopreventive activity. Another interesting aspect is the disparate activities mediated by structurally similar molecules, suggesting evaluation of a mixture could yield a better response with fewer side-effects than a single compound. 
